# Slow Cone Reflectance Changes during Bleaching Determined by Adaptive Optics Scanning Laser Ophthalmoscope in Living Human Eyes

**DOI:** 10.1371/journal.pone.0131485

**Published:** 2015-06-29

**Authors:** Masakazu Hirota, Suguru Miyagawa, Hiroyuki Kanda, Takao Endo, Tibor Karl Lohmann, Tomomitsu Miyoshi, Takeshi Morimoto, Takashi Fujikado

**Affiliations:** 1 Department of Applied Visual Science, Osaka University Graduate School of Medicine, Suita, Osaka, Japan; 2 Fundamental Technology Sec, R&D Department, Topcon Corporation, Itabashi, Tokyo, Japan; 3 Department of Ophthalmology, Osaka University Graduate School of Medicine, Suita, Osaka, Japan; 4 Department of Ophthalmology, University Hospital Aachen RWTH Aachen University, Aachen, Nordrhein-Westfalen, Germany; 5 Department of Integrative Physiology, Graduate School of Medicine& Frontier Biosciences Osaka University, Suita, Osaka, Japan; Shenzhen institutes of advanced technology, CHINA

## Abstract

To investigate the changes in the reflectance of human cone photoreceptors by an adaptive optics scanning laser ophthalmoscope (AO-SLO) during photobleaching. A custom-built AO-SLO with an observation light of 840-nm was used to measure the cone densities and the reflectance changes during bleaching by 630 nm red light emitting diodes. Measurements were made at 1° and 3° temporal to the fovea within an area of 1° × 1° in 8 eyes of 8 normal subjects. After dark-adaptation, images of the cone mosaics were recorded continuously for 5-min before, 5-min during, and after 5-min of light stimulation with a sampling rate of 5-Hz. The first positive peak (P1) was observed at 72.2 ± 15.0-s and a second positive peak (P2) at 257.5 ± 34.5-s at 1°. The increase of the reflectance of P1 was significantly larger at 1° (34.4 ± 13.9%) than at 3° (26.0 ± 10.5%; *P* = 0.03, Wilcoxon’s signed rank test). The average cone density at 1° (51123.13 ± 1401.23 cells/mm^2^) was significantly larger than that at 3° (30876.13 ± 1459.28 cells/mm^2^; *P* <0.001, Wilcoxon’s signed rank test). The changes in the reflectance of the cones during bleaching by red light had two peaks. The two peaks may be caused by regeneration of cone photopigment during bleaching.

## Introduction

Fundus reflection densitometry has been used to evaluate the kinetics of the bleaching and regeneration of the photopigments.[1,2] This technique is based on the changes of the retinal reflectance during and after visible light stimulation. The recovery time of photopigments is correlated with the bleaching time, the light intensity, wavelength, and the age in healthy subjects.[3,4] The results of earlier studies have shown that the recovery time was prolonged by retinal diseases.[5–7]

The conventional fundus reflection densitometer is not appropriate for studies on photopigment cycling because it is focused on the inner limiting membrane.[6,8–10]

The adaptive optics scanning laser ophthalmoscope (AO-SLO) enables confocal reflectance imaging of the cone mosaics in living human eyes.[11–16] Therefore, direct information on the photoreceptors can be obtained.

The retinal intrinsic signal represents the reflectance ofinfrared light from the retina, and the reflectance changes during and aftervisible light stimulationof the retina. The changes can be divided into fast (msec) and slow signals (sec to minutes).[17] The fast signals represent the neural activity elicited by the light stimulation, and the slow signals represent the processes related to cell swelling, metabolic changes, and /or hemodynamic changes secondary to the neural activation.[18] DeLint et al.[10] examined the reflectance changes induced by visible light stimulation of the retina using a conventional SLO and showed slow fluctuations of the reflected infrared light. Masella et al.[19] used an AO-SLO to measure the changes in the reflectance of photoreceptors in monkeys before and after photopigment bleaching with 514-nm light. The reflectance after bleaching measured by 794-nm showed fluctuations on the order of minutes.

The purpose of this study was to investigate the changes of the retinal intrinsic signal using AO-SLO in living human eyes, and to try to determine the possible mechanisms causing the fluctuations of the intrinsic signals.

## Materials and Methods

### Subjects

Eight right eyes of 8 young healthy adults whose mean age was 25.6 ± 3.7 years with a range of 21 to 33 years were studied. The mean refractive error (spherical equivalent) of the right eyes was -2.50 ± 1.97 diopters (D). All subjects had a comprehensive ophthalmological examination including color vision tests (AO-HRR, Dvorine, and Ishihara; Farnsworth dichotomous D 15 test). The best-corrected visual acuity (BCVA) was measured before the experiment to verify that they had normal visual acuity.

The right eye of each subject was dilated with 1.0% tropicamide and 1.0% phenylephrine hydrochloride, and each subject wore a soft contact lens (Menicon 1 day, Menicon Co., Ltd. Nagoya, Japan.) to correct the refractive error.

Because the ocular higher-order aberrations depend on tear film stability,[20] artificial tears (Soft Santear, Santen Pharmaceutial Co., Ltd. Osaka, Japan) were dropped on the corneas to prevent the reduction of tear film stability.[21,22] The room air conditioning was switched off during the experiments.[23,24]

A written informed consent was obtained from the subjects after the nature and possible complications of the study were explained. This investigation adheres to the tenets of the World Medical Association Declaration of Helsinki. The experimental protocol and the consent procedure were approved by the Institutional Review Board of the Osaka University Medical School. (14036–4)

### Adaptive optics scanning laser ophthalmoscope (AO-SLO)

A new model of a custom-built AO-SLO (Topcon, Itabashi, Japan) was used to measure the density of cone photoreceptors and the intrinsic retinal signals. A detailed description of the original AO-SLO has been reported.[25]

The ocular aberrations were measured with a Shack-Hartmann wavefront sensor at 30-Hz. The aberrations were corrected with a 10-Hz feedback loop with a 97-channel deformable mirror. After the wavefront corrections were made, the retinal images were acquired by illuminating the retina with a super-luminescent diode (SLD, Superlum Diodes, Ltd. Co. Cork, Ireland) with a lambda_max_ at 840-nm and a 12-nm bandwidth at half-amplitude. The output TIFF images consisted of 512 × 512 pixels with a 12-bit gray scale gradation. A star-burst fixation target was displayed on a white organic electroluminescent board with a size of 0.95°. The sampling rate of the changes of reflectance was 5-Hz.

A 1° × 1° area of the retina was photographed at 1° and 3° temporal to the fovea to calculate the cone density and the reflectance changes during and after the photobleaching. A high-pass optical filter was used to block light of wavelengths <750-nm to prevent the bleaching red light emitting diode (LED) from affecting the AO-SLO images created by infrared light.

### Photobleaching

A red LED with a wavelength of 630-nm, bandwidth of 8-nm, illumination angle of 120°, luminance of 3600 cd/m^2^ was used for photobleaching. Twenty-one red LEDs (diameter: 8-cm) were arranged circumferentially in front of the right eye and centered on the corneal apex. The retinal illuminance at an eccentricity of 1° and 3° was estimated using a model eye (axial length: 24.0-mm). The pupil diameter was restricted to 6.0-mm, and a pinhole (diameter 0.95°) was set in front of the focal plane of the model eye to block stray light.

### Experimental Protocol

After mydriasis and 10-min of dark-adaptation, the images of the cone mosaics were recorded continuously for 5-min in the dark, then for 5-min while bleaching with red light from the red LEDs, and then for 5-min after the cessation of bleacing. The measurements were made in a dark room with an ambient illuminance of 0.01-lx. The photostimulation period was set to 5-min to measure the reflectance changes while the photopigment completely bleached.[26]

Subject 2 underwent the same experiment one month after the first experiment to determine the repeatability of the changes of the cone reflectance induced by the light stimulation.

### Data Analyses

The noisy images caused by fluctuations of fixation or increases of aberrations were excluded from the analyses, and the suitable images were averaged. The cone densities were determined by an automatic detection algorithm [Miyagawa S, ARVO E-Abstract B0150, 2014].

The average intensity of the reflected light in a retinal area of 1° × 1° was computed by the ImageJ software (Rasband W, NIH, USA), and the average intensity of the reflected light before light stimulation was set as the baseline. A change in the reflectance was calculated as the average intensity of the reflected light during and after light stimulation divided by that of the baseline.

Changes in individual cone reflectances were also measured in Subject 2 to confirm the reflectance change in the 1° × 1° area originated from the cone photoreceptors. Fifty adjacent cones at an eccentricity of 1° temporal from the fovea were selected, and the reflectance was measured and averaged. In this procedure, the bright cone cells with saturated value of reflectance >3000 Grey Scale Value (GSV) were excluded. A low-pass filter was used to identify the peaks of the cone reflectance using Origin Pro 8.1J (OriginLab Co., Northampton, MA). The cut-off frequency was set at 0.008-Hz which displayed one-cycle at 60-sec. The noisy data caused by blinking or eye movements were deleted and linearly interpolated.

The time to the peak and the reflectance at the peak were measured for the first positive peak (P1), the first negative trough (N1), and the second positive peak (P2) during bleaching. The recovery time, i.e., time for the reflectance to decrease to the baseline value after the cessation of the light stimulation, was also measured.

### Statistical analyses

The differences in the cone densities, peak times, and reflectance changes between 1° and 3° were analyzed by Wilcoxon’s signed rank test with StatView 5.0 (HULINKS, Inc., Tokyo, Japan). The significance of the differences in the cone reflectances of P1, N1, and P2 were determined at the same eccentricity by Scheffé test with StatView 5.0. The significance level was set at *P* <0.05.

## Results

### Cone Density

The cone density at 1° was 51123.1 ± 1401.2 cells/mm^2^ which was significantly higher than that at 3° of 30876.1 ± 1459.3 cells/mm^2^ (*P* < 0.001, Wilcoxon’s signed rank test; Table 1).

**Table 1:** Cone density at 1- degree and 3-degree retinal eccentricity.

	Cone density (cells/mm^2^)	
Subject	1-degree	3-degrees
1	52984	31725
2	54654	31409
3	52179	30246
4	51586	31335
5	52443	30547
6	52436	28328
7	49734	30081
8	51969	33338
Ave	51123.13^***^	30876.13^***^
SD	1401.23	1459.28

***: *P* <0.001, Wilcoxon’s signed rank test.

### Retinal Illuminance Estimated in Model Eye

The retinal illuminance was 3.3 × 10^6^ td at both at 1° and 3° in the model eye. Thus, both retinal illuminances were well below the safety limit of the ANSI criterion.

### Changes of Reflectance during and after Bleaching

**Fig 1:**
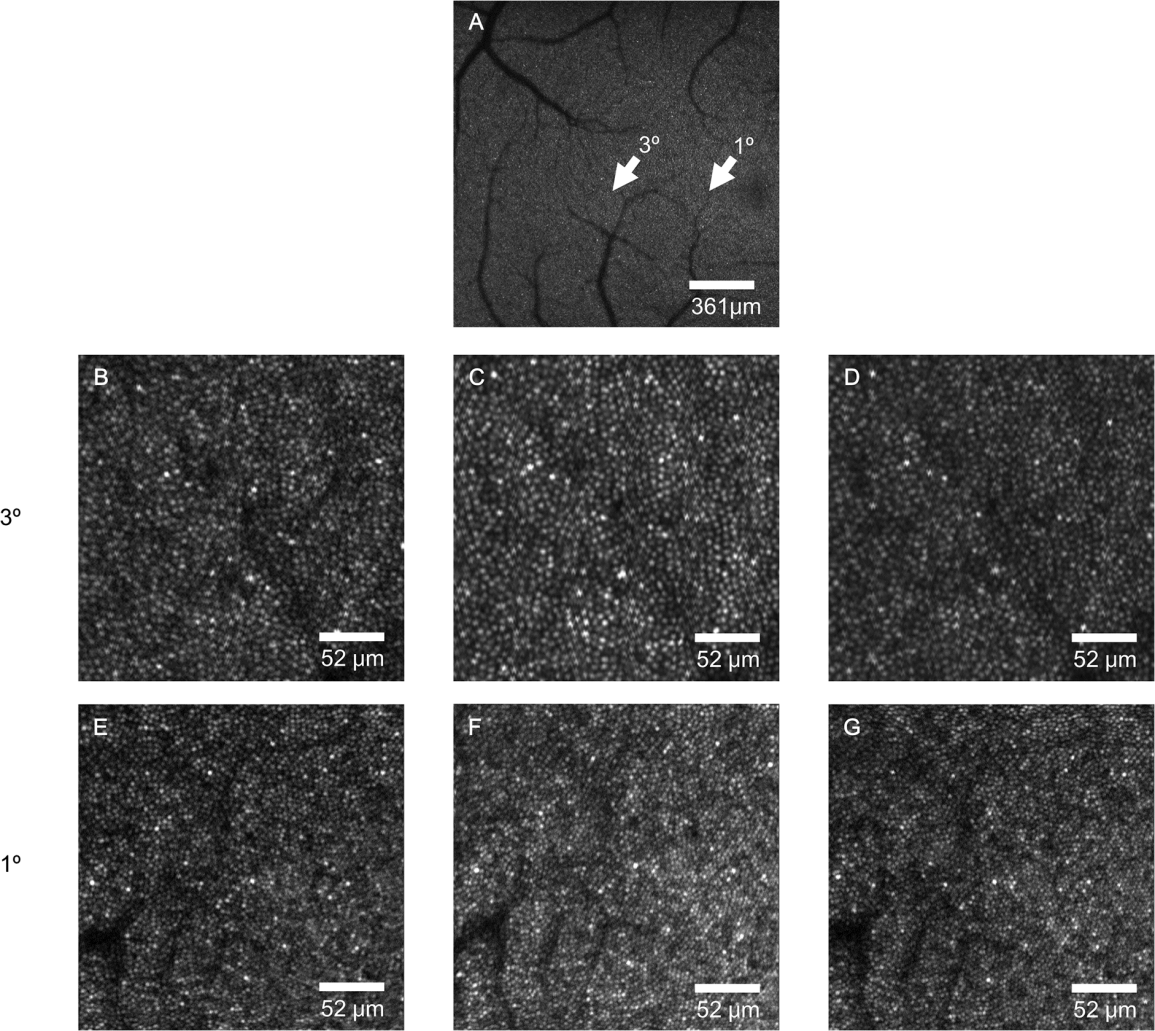
Images of photoreceptors in the right eye of subject 2 taken by AO-SLO. A: The AO-SLO image with low magnification (7° * 7°) with a center 3° temporal from the fovea. B-D: The AO-SLO image with high magnification (1° * 1°) at 3° temporal from the fovea as indicated in A. E-G: The AO-SLO image with high magnification (1° * 1°) at 1° temporal from the fovea as indicated in A. B and E: Images taken before light stimulation. C and F: Images taken during light stimulation (72-s after the onset of light stimulation). D and G: Images taken 60-s after the cessation of light stimulation.

**Fig 2:**
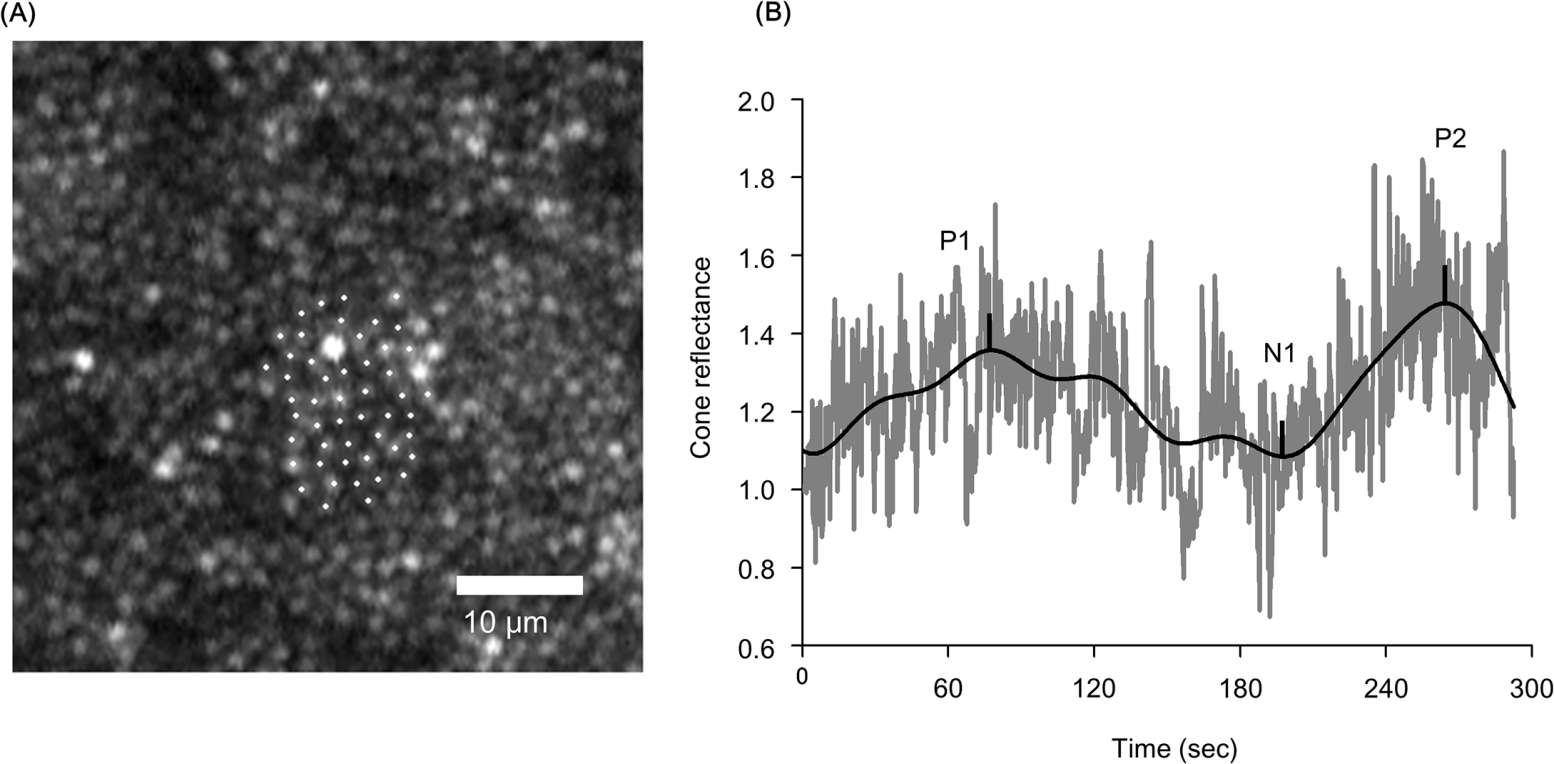
Individual cone reflectance changes during light stimulation. A. AO-SLO image with high magnification (1° * 1°) at 1° temporal from the fovea in Subject 2. White dots represent the 50 cones in which reflectance changes were measured. B. Average of the individual cone reflectance changes during light stimulation. The dark gray lines indicate the raw data. The black line shows the low-pass filtered data at 0.008-Hz. The average cone reflectance changes had two peaks. P1, first positive peak; N1, first negative trough; P2, second positive peak.

The cone reflectances increased during bleaching and decreased to the baseline level after cessation of bleaching (Fig 1). In Subject 2, the average reflectance of 50 adjacent cones at 1° temporal from the fovea increased during light stimulation and fluctuated during bleaching. The first positive peak was at 76.6-s and the second positive peak was at 261.3-s after the onset of light stimulation (Fig 2).

**Fig 3:**
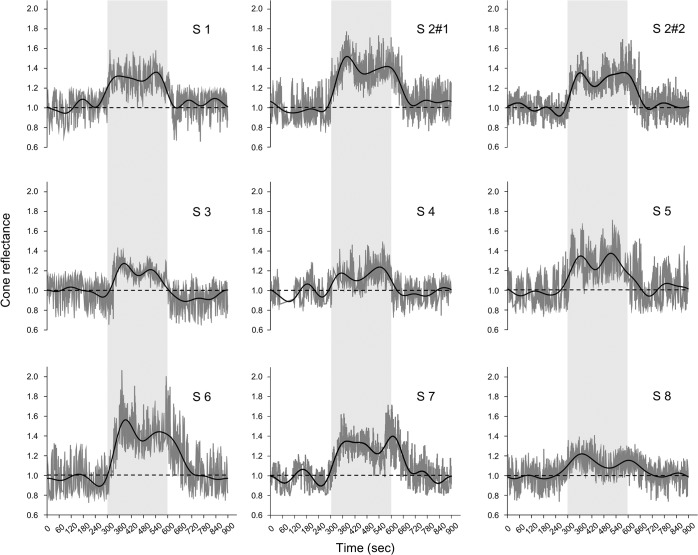
Reflectance changes at 1° temporal from the fovea. The dark gray lines indicate the reflectance changes at 1° temporal from the fovea. The black line shows the low-pass filtered data at 0.008-Hz. The dotted line indicates the baseline. The light gray zone indicates the period of light stimulation. Cone reflectances increased during light stimulation and had two peaks in all subjects. Subject 2 underwent the repeatability experiments (S2 #1 and S2 #2).

**Fig 4:**
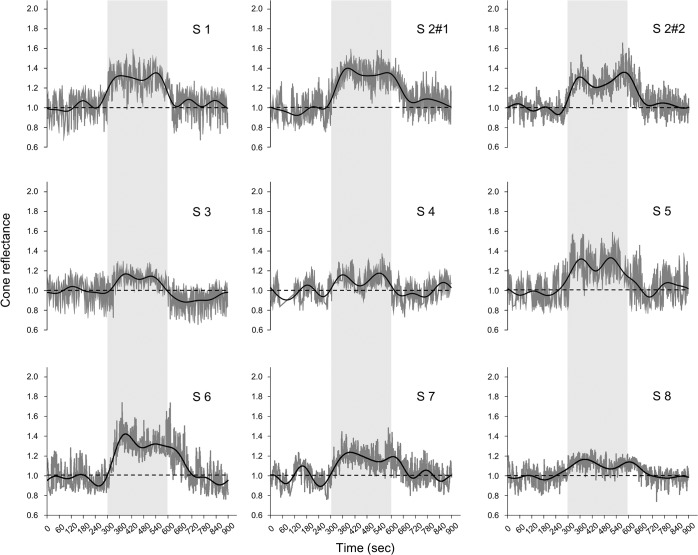
Reflectance changes at 3° temporal from the fovea. The dark gray lines indicate the reflectance changes at 3° temporal from the fovea. The black line shows the low-pass filtered data at 0.008-Hz. The dotted line indicates the baseline. The light gray zone indicates the period of light stimulation. Cone reflectance increases during light stimulation and has two peaks in all subjects. Subject 2 underwent the repeatability experiments (S2 #1 and S2 #2).

The reflectance of the AO-SLO images of the 1° × 1° area at 1° increased after the onset of light stimulation and had two peaks during the light stimulation in all 8 subjects (Fig 3). The average reflectance of the 1°×1° retinal area at 3° also increased after the onset of light stimulation and had two peaks during the light stimulation in all 8 subjects (Fig 4).

The average reflectance of the AO-SLO image at the 1° × 1° retinal area increased after the onset of light stimulation and reached the first peak (T_P1_) at 72.2 ± 15.0-s after the beginning of light stimulation with an increase of reflectance (ΔI) of 0.34 ± 0.13 at an eccentrisity of 1° and a T_P1_ of 79.6 ± 15.0-s with a ΔI of 0.26 ± 0.10 at an eccentricity of 3°. The T_P1_ at 1° was significantly shorter than that at 3° (*P* = 0.02, Wilcoxon’s signed rank test) and the ΔI at 1° was significantly higher than that at 3° (*P* = 0.03, Wilcoxon’s signed rank test).

After P1, the reflectance decreased and reached the first trough (T_N1_) at 170.8 ± 33.7-s with a ΔI of 0.21 ± 0.10 at an eccentricity of 1°, and a T_N1_ of 177.0 ± 33.1-s with a ΔI of 0.17 ± 0.10 at an eccentricity of 3°. The T_N1_ at 1° was significantly shorter than that at 3° (*P* = 0.02, Wilcoxon’s signed rank test).

**Fig 5:**
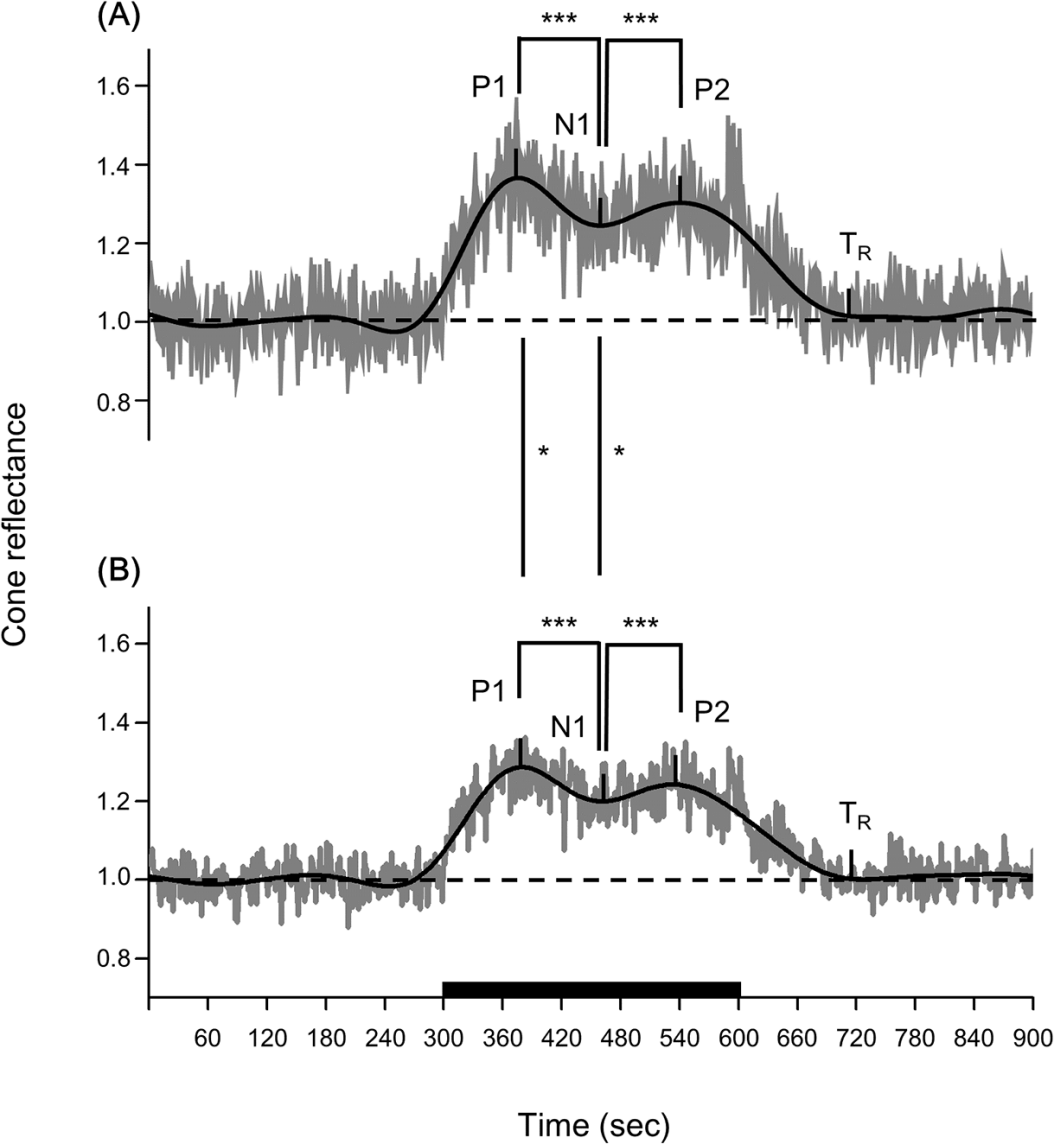
Average of reflectance changes. A. The average of reflectance changes at 1° temporal from the fovea. B. The average of reflectan B. ce changes at 3° temporal from the fovea. P1: first positive peak. N1: first negative trough. P2: second positive peak. T_R_: recovery time. *: *P* = 0.02, Wilcoxon’s signed rank test. ***: *P* <0.001, Scheffé test.

After N1, the reflectance increased to reach a second positive peak (P2) with a peak time (T_P2_) of 257.5 ± 34.5-s with a ΔI of 0.31 ± 0.10 at an eccentricity of 1° and a T_P2_ of 262.9 ± 39.7-s with a ΔI of 0.24 ± 0.09 at an eccentricity of 3°. The ΔI at 1° was significantly larger than that at 3° (*P* = 0.01, Wilcoxon’s signed rank test; Fig 5, Table 2).

**Table 2:** Peak time and change of cone reflectance (ΔI).

	P1		N1		P2	
Retinal eccentricity	Peak time (s)	Change of reflectance	Peak time (s)	Change of reflectance	Peak time (s)	Change of reflectance
1-degree	72.2 ± 15.0	0.34 ± 0.13	170.8 ± 33.7	0.21 ± 0.10	257.5 ± 34.5	0.31 ± 0.10
3-degrees	79.6 ± 15.0	0.26 ± 0.10	177.0 ± 33.1	0.17 ± 0.10	262.9 ± 39.7	0.24 ± 0.09

The ΔI of P1 and P2 were significantly larger than that of N1 at both retinal eccentricities (*P* <0.001, Scheffé test; Fig 5, Table 2).

After the cessation of light stimulation, the reflectance decreased and reached the baseline (T_R_) at 109.9 ± 45.8-s at 1° and 111.1 ± 51.3-s at 3° (Fig 5).

## Discussion

### Reflectance Changes detected by Near-Infrared Observation Light during Visible Light Stimulation

We detected an increase in the reflectance of the retina during visible light stimulation of the retina by AO-SLO (Fig 1). The average reflectance of individual cones increased during the visible light stimulation (Fig 2). These results indicate that the increase of reflectance is due to changes in the cone photoreceptors.

Because the cone mosaics observed by AO-SLO originate mainly from the cone outer segments,[27] the increase of reflectance can be attributed to the changes in reflectances of the cone outer segments. The results of animal studies showed that near-infrared light is able to monitor structural changes in the cone outer segments and also changes in the interphotoreceptor matrix.[17,28–30]

Our results are consistent with the data of Roorda et al who showed that the cone reflectance was increased by the light stimulation as determined by AO-SLO.[12]

### Biphasic Changes of Reflectance during Light Stimulation

The time course of the reflectance changes at the parafoveal retinal area had a biphasic pattern during visible light stimulation (Figs 3 and 4). The repeat measurement of Subject 2 showed good reproducibility (Figs 3 and 4). The average reflectance of the individual cones also had a biphasic pattern during bleaching (Fig 2). These results suggest that the biphasic changes of reflectance detected by infrared light during visible light stimulation were caused by changes of the cone outer segments during photopigment bleaching.

The first positive peak (P1) was at 72.2 ± 15.0-s at 1° and 79.6 ± 15.0-s at 3° and the second positive peak (P2) at 257.5 ± 34.5-s at 1° and 262.9 ± 39.7-s at 3° (Fig 5, Table 2). DeLint et al.[10] examined the reflectance changes of living human retinas in a 2.5° × 2.5° square around the fovea by near-infrared light (790-nm) with a conventional SLO during visible-light stimulation (560-nm) for 16 minutes. They showed an increase of reflectance with a peak a couple of minutes after the beginning of visible light stimulation (Fig 2 of DeLint et al). Their results are consistent with our results although their data were quite scattered.

Burkhardt[26] examined the reflectance changes of isolated turtle retinas using near-infrared observation light during photobleaching with 630-nm light for 5.3-min. He reported that the retinal reflectance takes about 100 to 120-s until it reaches the first positive peak (Fig 3 of Burkhardt).

Our data together with the results of these two studies suggest that the reflectance of near-infrared light initially increases, then decrease, and increases again during bleaching.

Ala-Laurila et al. demonstrated that the concentration of all-*trans* retinol reaches a maximum at 60- to 120-s after light stimulation in the salamander’s cones by microfluorometry.[31] They reported that the concentration of all-*trans* retinol decreased after reaching the maximum. The results of earlier biochemical studies indicated that the photopigments supply to the cone photoreceptor is through the cone specific visual cycle in humans and animals.[32–39] Therefore, we suggest that the cone reflectance significantly decrease after the first positive peak because the photopigments were supplied to the cone photoreceptors. The cone reflectance significantly increased again by the bleaching of the photopigments.

### Reflectance Changes after Cessation of Light Stimulation

The time course of reflectance changes at the parafoveal area had a monophasic decrease to baseline at 109.9 ± 45.8-s at 1° and at 111.1 ± 51.3-s at 3° after the cessation of light stimulation (Fig 5). The previous reports of densitometry and electroretinography showed that the recovery time was about 2-min after full bleaching[4,6,40,41], which was close to our recovery time. Recently, Masella et al. also demonstrated by AO-SLO that slow reflectance changes occur in the monkey’s cone and rod photoreceptors after the cessation of light stimulation.[19] Although the reflectance changes detected by near-infrared light does not directly indicate the absorption by the photopigments, these results suggest that the slow reflectance changes after bleaching may be related to the recovery of the photopigments. The molecular mechanism how the slow reflectance changes are induced in the near-infrared light after bleaching should be clarified by animal experiments in future.

### Differences of Cone Reflectance Changes by Retinal Eccentricity

The results show that the cone reflectance changes at 1° were significantly greater than that at 3°. Because the retinal illuminance at 1° and at 3° were identical as measured in the model eye, the differences of the cone reflectance changes are most likely due to the cone density. We have calculated the cone density directly and have found a significant difference of cone density between 1° and 3° (Table 1).

Kazato et al.[42] showed that the retinal reflectance at the fovea was higher than that at the parafovea which is consistent with our results. They suggested that the retinal reflectance depended on the cone density as we have.

## Conclusions

The reflectance of cone photoreceptors observed by infrared light showed two peaks during bleaching by red light. The two peaks are probably caused by changes in the cone outer segments that are related to the regeneration of the cone photopigments after bleaching.
